# A cluster-randomized trial study on effectiveness of health education based intervention (HEBI) in improving flood disaster preparedness among community in Selangor, Malaysia: a study protocol

**DOI:** 10.1186/s12889-021-11719-3

**Published:** 2021-09-24

**Authors:** M. N. Mohd Tariq, Hayati Kadir Shahar, Mohd Rafee Baharudin, Sharifah Norkhadijah Syed Ismail, Rosliza Abdul Manaf, Md Said Salmiah, Jamilah Ahmad, Sri Ganesh Muthiah

**Affiliations:** 1 Department of Community Health, Faculty of Medicine and Health Sciences, Universiti Putra Malaysia, 43400 Serdang, Selangor Malaysia; 2 Malaysian Research Institute of Ageing (MyAgeing), Universiti Putra Malaysia, 43400 Serdang, Selangor Malaysia; 3 School of Communication, Universiti Sains Malaysia, Penang, Malaysia

**Keywords:** Disaster preparedness, Health education, Health belief model, Community

## Abstract

**Background:**

Flood disaster preparedness among the community seldom received attention. Necessary intervention must be taken to prevent the problem. Health Education Based Intervention (HEBI) was developed following the Health Belief Model, particularly in improving flood disaster preparedness among the community. The main objective of this study is to assess the effect of HEBI on improving flood disaster preparedness among the community in Selangor. This study aims to develop, implement, and evaluate the impact of health education-based intervention (HEBI) based on knowledge, skills, and preparedness to improve flood disaster preparedness among the community in Selangor.

**Method:**

A single-blind cluster randomized controlled trial will conduct at six districts in Selangor. Randomly selected respondents who fulfilled the inclusion criteria will be invited to participate in the study. Health education module based on Health Believed Theory will be delivered via health talks and videos coordinated by liaison officers. Data at three-time points at baseline, immediate, and 3 months post-intervention will be collected. A validated questionnaire will assess participants’ background characteristics, knowledge, skill, and preparedness on disaster preparedness and perception towards disaster. Descriptive and inferential statistics will be applied for data analysis using IBM Statistical Package for Social Sciences version 25. Longitudinal correlated data on knowledge, skills, preparedness, and perception score at baseline, immediate post-intervention, and 6 months post-intervention will be analyzed using Generalized Estimating Equations (GEE).

**Discussion:**

It is expected that knowledge, skills, preparedness, and flood disaster perception score are more significant in the intervention group than the control group, indicating the Health Education Based Intervention (HEBI).

**Trial registration:**

Thai Clinical Trial TCTR20200202002.

## Background

The definition of a disaster is an event that can cause disturbance and disruption of social activity and nation’s business, loss of life, damage to property, economic loss or environmental destruction, which goes beyond the society’s capability to overcome it and requires an extensive pooling of resources [1]. At the same time, a flood can be defined as an excessive volume of water that can inundate a wide area or property.

Therefore, a flood is recognized as a natural disaster. The flood can be linked to several floods, such as beach floods, flash floods, river floods, underground floods, and sewage floods [2, 3]. Beach flood occurs when storms or extreme weather combined with a high tide causing sea level to rise above the average level, forcing seawater to overflow into the land. Heavy rainfalls cause a flash flood on an area, which collects a large volume of water rapidly. Due to an inadequate drainage system or waste or segregated material blockage [3].

On the other hand, flood disaster preparedness across communities is rarely discussed, either by individuals, health care providers, or the government, before complications occur. Certain groups are unaware that disadvantaged people, such as women, children, and the elderly, need special consideration in disaster preparedness [4]. As a result, it would benefit the community’s existing disaster preparedness if this intervention is successful.

Some theories explain behavior and suggest developing an effective way to influence behavior, such as Health Belief Model theory, Ecological theory, Social Cognitive Theory, Knowledge, Attitude and Preparedness theory, and Precaution Adoption Process Model theory. In these theories, researchers are provided with a guide to understanding people’s behavior toward health promotion and identifying information needed to design an effective strategy. Other than that, researchers are also guided to provide insight on how to create a successful program. On the other hand, interventions based on a theoretical foundation are more effective and result in a successful public health intervention program [5]. The function of theories is to understand why people chose to agree and refused to participate in healthy behavior.

One of the most established and oldest conceptual frameworks of health behavior is The Health Belief Model (HBM). This model is applied to disaster preparedness efforts, mainly focusing on human behavior [6]. Preparedness efforts focus on changing human behaviors to reduce people’s risk and increase their ability to cope with hazard consequences. Health Belief Model was developed by focusing on the attitudes and beliefs of individuals. There are four main pillars in the Health Belief Model that representing the perceived threat and net benefits. In addition, they have perceived susceptibility, perceived severity, perceived usefulness, and perceived barriers. This model was introduced as auditing for people’s readiness to act. Additionally, cues to action would activate that readiness and stimulate overt behavior. On the other hand, the self-efficacy concept of inefficiently performing is a recent addition to the Health Belief Model [7].

**Fig. 1:**
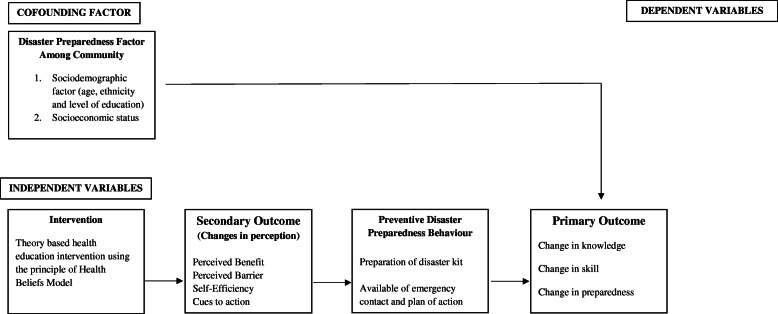
Conceptual framework using Health Belief Model on Flood Disaster Preparedness

This study will provide a platform for increasing knowledge and promoting correct skills and preparedness toward flood disaster preparedness. This study will also contribute to the body of knowledge on the preparation of flood disasters among the community. This study aims to develop, implement, and evaluate health education-based intervention (HEBI) based on knowledge, skills, and preparedness as the primary outcome and changing of disaster perception (Perceived Benefit, Perceived Barrier, Self-Efficiency, Cues to action) as secondary outcome using the Health Believes Theory Model to improve flood disaster preparedness among the community. The conceptual framework as in Fig. 1.

## Methods

### Trial registration

1. Name: Thai Clinical Trial
2. Trial Registration Number: TCTR20200202002
3. Date of Registration: 02 February 2020
4. Overall Recruitment Status: Recruiting
5. Study Start Date (First enrolment): 01 June 2020
6. Completion Date (Last subject, Last visit): 31 May 2023
7. Study Completion Date: 01 September 2023
8. URL of Trial Registry Record: https://www.thaiclinicaltrials.org/show/TCTR20200202002

### Study design and study location

This study is a two-armed, parallel, single-blinded, cluster-randomized trial. The cluster in this study is defined as a district in Selangor. There are two groups which are the intervention group and the control group. Both groups follow a similar design until the end of the study. The clusters and participants are blinded for the study. The intervention group will receive HEBI, while the control group will receive a non-communicable health program.

Selangor is one of 13 states in Malaysia, located on the west coast of the Malaysian Peninsular. Selangor covers 7930 km2 [8], with a total population of 6.47 million reports to the Department of Information Malaysia, ranking it the most populous state in Malaysia (19.9%). It has a population growth rate, 1.4% and a high level of urbanization (91.4%) [8]. In addition, it has the largest economy in Malaysia in terms of gross domestic product (GDP) contribution, contributing 22.4% of the total Malaysian GDP [8]. The ethnic composition consists of Malay, 52.9%, Chinese, 27.8%, Indian, 13.3%, and other ethnic groups.

The study will carry out in six districts in Selangor state. First, those districts are chosen based on similar backgrounds related to the flood disaster. Then, those districts are ranked based on floods every year and the severity of the disaster using the Department of Irrigation and Drainage Malaysia data. Based on the rank, district Hulu Langat, Hulu Selangor, Kuala Selangor, Sepang, Sabak Bernam and Gombak were included in this study. Then those districts are divided into intervention groups and control groups using a coin toss. As for respondents, they were selected from each household in those districts based on eligibility.

### Study population and respondent selection

The sampling population is the communities from flood-prone areas in Selangor that fulfilled the inclusion and exclusion criteria. An individual from each household selected from Hulu Langat, Hulu Selangor, Kuala Selangor, Sepang, Sabak Bernam, and Gombak in Selangor state met the inclusion and exclusion criteria. The inclusion criteria are a citizen and individual age 18 years old and above, and the exclusion criteria are literate individuals and physically handicapped status.

### Sample size estimation

The sample size (N) for this study was calculated by comparing the populations of two groups when the endpoint is quantitative. Using the formula by Lameshow and Lwanga (1990):

$$ N=\frac{{\left[{\mathrm{Z}}_{1-\sigma /2}\surd 2\mathrm{P}\left(1-\mathrm{P}\right)+{\mathrm{Z}}_{1-\beta}\surd {\mathrm{P}}_1\left(1-\mathrm{P}\right)+{\mathrm{P}}_2\left(1-{\mathrm{P}}_2\right)\right]}^2}{{\left({P}_1-{P}_2\right)}^2} $$

Where,

N = Sample size estimation

Z_1− σ/2_ = Standard error associated with 95% confidence interval 1.96

Z_1− β_ = Standard error associated with 80% power = 0.842

P_1 =_ Population proportion 1

P_2_ = Population proportion 2

P = P_1 +_ P_2_ / 2

Thus, the calculation below is based on study flood preparedness [9]:

The proportion of follow-up for flood preparedness in the intervention group.

P_1_ = 79.6% = 0.79.

Proportion of follow-up for flood preparedness in control group.

$$ {\mathrm{P}}_2=40.2\%=0.40 $$

$$ \mathrm{P}={\mathrm{P}}_{1+}{\mathrm{P}}_{2/}2=\left(0.79+0.40\right)/2=0.59 $$

$$ N=\frac{{\left[1.96\surd 2(0.59)\left(1-0.59\right)+0.84\surd 0.79\left(1-0.79\right)+0.40\left(1-0.40\right)\right]}^2}{{\left(0.79-0.40\right)}^2} $$

$$ N=3.53/0.15 $$

$$ N=24 $$

Taking into account comparison 2 groups.

$$ N=48 $$

Taking into account the design effect = 48 x [1 + (m-1) ICC].

Where m = average cluster size, ICC = intracluster correlation coefficient.

m = 31 [10], ICC = 0.005 based on adjusted ICC for individual- and cluster-level characteristics [10].

Thus, 48 × [1 + (31 – 1)0.005] = 55.2

Adjusting 34% attrition rate [11].

55.2 / 0.66 = 84 final sample sizes estimated for both intervention and control group.

### Sampling method and subject recruitment

**Fig. 2:**
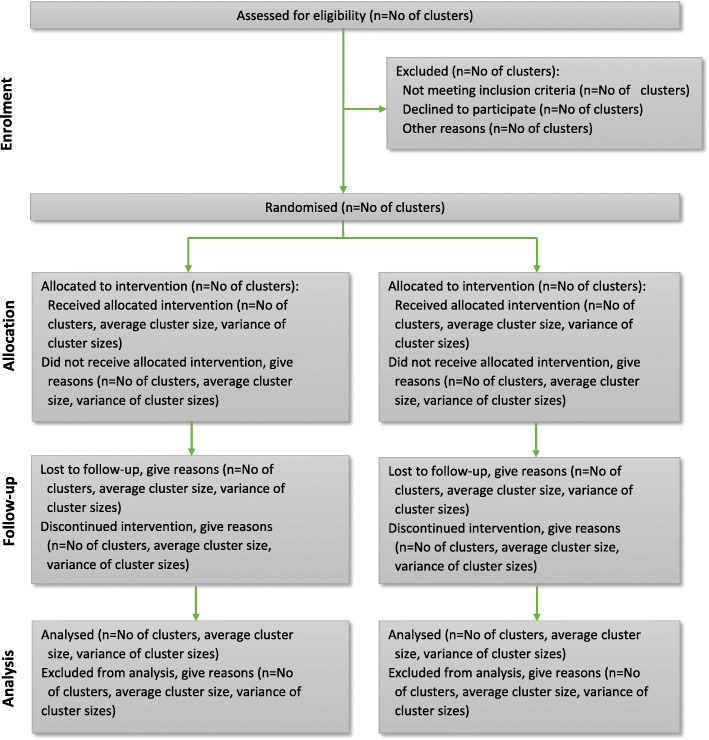
CONSORT extension for Cluster Trials

Randomization of the participants will conduct based on the district they belong to. The six districts chosen earlier were randomly assigned into the intervention and control groups with 1:1 allocation. Thus, three districts belong to the intervention group, and three are used in the control group. Figure 2 shows the procedures involved in the recruitment of subjects in this study.

In each area, the participant’s name list is a number code by the researcher. Only the researcher has access to the names and codes and is solely responsible for all the records’ safekeeping and confidentiality. Concealment of allocation or a “third-party” assignment will use. The list of these codes without the individual’s names will be sent to a research assistant (RA) to produce a computer-generated randomization list of the participants. The randomization software used is from the web page of Research Randomizer [12].

Participants will recruit based on the districts they represent to prevent communication between the intervention and the control groups and minimize the contamination. For example, if District A has been chosen in the intervention group, the communities in District A will include in the intervention group. As for District B, the communities in district B will consist of the control group. A simple random sampling will identify the respondents based on the household list in six selected districts during the recruitment process. Those who are eligible will be invited to participate in the study.

### Data collection method

The delivery of the intervention is design to address the limitations that occurred during the COVID-19 pandemic. The intervention and data collection will be carried out at the study site by liaison officers. The researcher can use the module and communicate with the liaison officers about the intervention’s flow and data collection. Respondents will give their consent through consent forms. Subsequently, respondents will be provided with a questionnaire to fill out, utilizing baseline data for the study (T0). Immediately upon completion of the health intervention, respondents will be provided with a questionnaire to respond to the immediate post-intervention assessment (T1). Knowledge, skills, and preparedness outcomes will be assessed at this stage. Six months after completing the health education intervention, respondents would be reassessed on the same effects as knowledge, skills, and preparedness (T2).

### Intervention

The intervention arm will receive the Health Education Based Intervention module (HEBI) developed based on the Health Belief Model Theory as in Table 1, consisting of:

i. The two-hour educational talk will be conducted to all participants, including the importance and benefit of disaster preparedness, difficulties during the disaster, joint issues, and steps to success.
ii. Educational disaster preparedness video in which the benefits of disaster preparedness will be introduced and demonstrated.
iii. Booklets describing the importance of disaster preparedness, written, and published by the Ministry of Health Malaysia, were distributed. It contains practical during disaster and management of ordinary during disaster problem problems.
iv. Brainstorming sessions with the researcher also provide for the community to overcome any individual problem.
v. The community supports a disaster preparedness support group in which people can contact them personally by phone during the intervention program.

**Table 1:** Summary of the Intervention Activities

Stage	HEBI Components	Theoretical Model Constructs	What and How It Is Delivered
Knowledge	i. Group education (health talk and group discussion) ii. Surrogate experimental learning (Brochure, flyer, poster, video)	i. Perceived Benefit ii. Perceived Barrier iii. Self-Efficiency iv. Perceived susceptibility	i. General knowledge about disaster preparedness will be assessed, and all material will be disseminated during the intervention. ii. Perceived benefit and perceived benefit were assessed during the discussion about the disaster preparedness barrier and the benefit of preparedness. iii. A baseline questionnaire will assess every individual before intervention is programmed. iv. The intention of the preparedness will be assessed.
Skills	i. Interactive learning with the community (brainstorming)	i. Cues to action	i. Information related to the advantage and disadvantages of preparedness. ii. Information about disaster preparedness was explaining. iii. Information on HEBI intervention how to prepare individuals for disaster.
Preparedness and implementation	i. First Aid demonstration ii. Field experimental learning with the group (field visit to disaster area at the community level, mapping the vulnerability area at the community level, and revising emergency plans at community level)	i. Cues to action	i. Preparation of the disaster kit was assessed before and after the intervention. ii. Available of emergency contact and plan of action after intervention

#### Control

Data will be obtained in the same way for the control group as it was for the intervention group: baseline (T0), immediate post placebo (T1), and 6 months post placebo (T2). In addition, a non-communicable disease health talk will be provided to the placebo group.

### Outcome assessment

The questionnaire has seven socio-demographic, socio-economic, personal characteristics, knowledge, skills, preparedness, and perceived disaster preparedness. Socio-demographic has six items, socio-economic status has five items, personal characteristic has two items, knowledge has nine items, skills have ten items, preparedness has nine items.

Prompted responses to the nine questions on knowledge were considered for calculating knowledge scores. The total score on knowledge was calculated by combining scores from 9 questions. Score 1 was given if the answer was ‘yes’ and no negative marking was given for ‘no.’ The maximum possible score for the knowledge part was 9. Good knowledge refers to respondents who have scored more than 50% of knowledge questions, and low knowledge refers to respondents who have achieved less than 50% of knowledge questions [13, 14]..

For skills score, each respondent was asked 10-item questions related to their skills towards disaster preparedness. The maximum possible score for the attitude section was 10. Negative skills represent those respondents who have scored less than 50% from skills questions, and positive skills indicate respondents who have achieved greater than or equal to 50% from skills questions [13, 14].

Each respondent was asked if they prepared any particular action to help create better disaster preparedness. Scores were given accordingly, and no negative marking was given. The maximum score possible was 9 [13, 14].

Perceived disaster preparedness questionnaire adapted from a previous study done in Turkey [15]. This questionnaire extracted six perceived which are Self-efficacy (10 items), Cues to action (5 items), perceived susceptibility (6 items), perceived barriers (14 items), perceived benefits (6 items), and perceived severity (4 items). Cronbach’s alpha coefficient for the subscales ranged from 0.90 to 0.74. Therefore, the GDPB scale based on the HBM was found to be a valid and reliable tool.

Respondents completed scales assessing “susceptibility”, “severity”, “benefits”, “barriers”, “self-efficacy” and “cues to action”. All items were scored on a five-point Likert scale from 1 (strongly disagree) to 5 (strongly agree). All subscales measured General Disaster Preparedness Belief (GDPB). Negatively worded statements (4,6,8,9,17-30,31,35,37,38,42,44) were used, the scores on the items were reverse-scored so that a higher score represented more positive disaster preparedness belief. Total score for each item for susceptibility (30), severity (20), benefits (30), barriers (70), self-efficacy (40), and cues to action (50). Responses were scored and categorized as high (75th quartile), moderate (75–25th quartiles), and low (25th quartile).

The GDPB score measured respondent’s positive disaster preparedness beliefs. It was computed by summing up the six subscales (Self Efficacy + Cues to action + Perceived susceptibility + Perceived low barrier (items were reverse scaled) + Perceived benefits + Perceived severity). Thus, it measured six dimensions with 45 items.

### Project monitoring and quality control

#### Validity

Threats to internal validity were taken into consideration in this research. The main threats that are controlled for intervention infidelity. The intervention module, HEBI, will be developed in a way that others can replicate it. It comprises four delivery techniques: health talk, group discussion, demonstration session, and group discussion.

Other threats include threats by history. In addition, there are unplan events between interventions, such as the control group participant knowing some intervention group participants when learning the intervention module’s content and knowledge on disaster preparedness.

Maturation means the participants change during the trial, such as having an emotional event during the answer baseline questionnaire, to make the wrong answer. For example, during post-intervention data collection, having a dynamic fit and answer the questionnaire correctly and confidently. However, they result in an erroneous increase in scores from the test. Thus, intervening’s the actual effect. To counter this problem, get consent before answering the pre-test question and not force potential personnel to participate in the study. There may also be selection bias in choosing which organization will be in the intervention or control groups.

In terms of questionnaire validity, four experts in public health and one expert in medical will do the content validation. Content validity ratio will calculate for each item, with each item obtained a minimum value of 0.5.

The questionnaire will translate to Bahasa Melayu, the researcher’s national language, four experts in public health, and one expert in medical. For face validity, first, the participants evaluate the questionnaire measures what it intends to measure in terms of the questionnaire’s comprehensiveness and clarity. Secondly, whether the questionnaire is simple, easily understood, inappropriate, redundant, or missing items and how likely the questionnaire was to address the research objectives. Third, whether the flow, arrangement, and wording of the questionnaire are reliable. For reliability, the questionnaire was pre-test to ensure reliability before the final version is used.

## Ethical consideration

Ethical clearance obtained from Ethics Committee for Research Involving Human Subjects Universiti Putra Malaysia (Jawatankuasa Etika Universiti Untuk Penyelidikan Melibatkan Manusia (JKEUPM) with JKE approval number UPM/TNCPI/RMC/JKEUPM/1.4.18.2 (JKEUPM). Besides that, written informed consent from the respondents has obtained before the study.

## Data analysis

### Data entry and analysis

Respondents’ responses to distributed questions will be used to enter data. The research assistant will document the response into IBM SPSS Version 25 (Statistical Package for Social Sciences). Before the final review, data cleaning will be performed to look for any missing data or outliers. Then, the data will be translated to long-form for analysis using the Generalized Estimating Equation (GEE). Descriptive and inferential statistics will be used to analyze the data. In descriptive statistics, continuous data will be reported in either a mean with standard deviations or a median with an interquartile range. Categorical data will be represented in percentages, while continuous data will be reported in either a mean with standard deviations or a median with an interquartile range. The histogram, skewness over the standard error of skewness, and statistical tests of Kolmogorov-Smirnov and Shapiro-Wilk normality tests will be used to screen for normality. If the normality test conditions are met, parametric tests will be used to continue the analysis; otherwise, non-parametric equivalents will be used. A significant alpha level of 0.05 will be used for inferential analysis, and *P*-values will be stated as two-sided.

### Missing data analysis

In this analysis, the purpose to treat concept (ITT) will be applied. Thus, when respondents’ final results are compared to their initially allocated party, regardless of care allocation, loss to follow up, or violation of the original protocol, they intend to treat the study. As a result, all missing data will be examined in this analysis, as the ITT concept can only be implemented when all respondents’ data is available.

The ability to perform ITT will be jeopardized if data is missing, and its conclusions will be undermined. Before the start of the research, strategies for dealing with missing data were devised. The amount of missing data explored, the trends involved, and variables associated with missingness would be the methods of approaching them. First, determine the sensitivity of the data. The primary analysis will be performed using the multiple imputation process, followed by sensitivity analysis. The aim to treat definition (ITT) will be used in this research. Intention to treat studies compares respondents’ final results to their initially assigned party, regardless of treatment allocation, follow-up loss, or the original protocol’s violation.

As a result, all missing data will be analyzed as part of this study since the ITT definition can only be applied if all respondents’ data is available. If data is lacking, the ability to execute ITT will be jeopardized, and the conclusions drawn from it will be undermined as well. Strategies for coping with missing data were formulated before the start of the study. The methods for approaching them would be investigating the amount of missing data, the patterns involved, and the variables associated with missingness. The primary analysis will be done with multiple imputations, and then a sensitivity analysis will be done to see how stable the conclusions are with and without missing data. Conclusions’ robustness when missing data is present and when it is not.

### Analysis of baseline variables

This analysis is being carried out to address the study’s first objective. The data will first be tested for normality using graphical and statistical techniques. The main objectives of analyzing baseline variables are to demonstrate comparability of respondents’ characteristics between treatment groups, to provide for covariate-adjusted analysis, which refines the overall treatment effect by taking into account baseline characteristics that are correlated to the outcome, and to set the context for subgroup analysis, which examines treatment differences on a more detailed level (Pocock et al., 2002). The chi-square test will be used for categorical data, while the independent T-test or Mann-Whitney U test will be used for continuous data.

### Generalized estimating equation (GEE)

The data from the sample will be calculated longitudinally to address the second and third hypotheses (in repeated measures). Comparing the variations in results between and within treatment groups over time points when adjusting for covariates, the Generalized Estimating Equation (GEE) will be used.

## Discussion

The expected outcome can be divided into future researchers, public health practices, and policymakers. First, some of the theory components and additional variables such as the level of Protective Behavior (Kirschenbaum, 2002) can be evaluated in a future study with pre- and post-intervention. Second, the qualitative analysis should be included in future studies to get feedback and develop the material. Focus group discussions, for example, will highlight components of the intervention that will be beneficial and components that need to be improved using qualitative design. Third, the study’s follow-up period should be longer and with more intervals to see the results after a year and prevent the effects of Pandemic COVID-19 and fasting month.

The HEBI module is beautifully developed and The HBM theory is used to indicate that a person’s belief in a personal threat of illness or disease, combined with a person’s belief in the efficacy of the prescribed health behavior or action, would predict the probability of the person adopting the behavior. This intervention shall be expanded to other agency examples, governmental tertiary institutions, and others state. The HEBI Module intervention can also be implemented at the National level disaster preparedness program as part of the counselor module and expanded into other ministry examples in the Ministry of Health Malaysia. With enough resources, the intervention shall repeatedly motivate the community to prepare, make a repeated attempt, and eventually succeed.

For policymakers such as the Malaysia National Disaster Preparedness Agency (NADMA), Ministry of Health Malaysia (MOH), this HEBI module can also be one of the modules that can be used together with existing modules to ensure that each perception can be identified and can change knowledge, skills and at the same time make the community itself prepared in the face of flood disasters.

## Conclusion

As we can see, a flood disaster could not be prevented by a human. However, we can minimize the wounded and forecast its occurrence. Some of the frequent natural disasters are landslides, earthquakes, tsunami, hurricanes, and others.

According to a previous study, a disaster is caused by human activity. Furthermore, immoral behaviors contribute to nature’s destruction as well. The obliteration of forests, air, sea, and others is becoming excessive in the modern era. For example, individuals involved in logging do not consider cutting trees. Therefore, better to be safe than sorry, and very important for each individual in the community must ready with the knowledge, skills, and preparedness toward disaster preparedness.

## Data Availability

The datasets used and analyzed during the current study are available from the corresponding author on reasonable request. However, any publications containing the results of this study have not been published or submitted to any journal.

## Abbreviations

HEBI: Health Education Based Intervention
GEE: Generalized Estimating Equations
